# Enhancement of Domain Wall Pinning in High-Temperature Resistant Sm_2_Co_17_ Type Magnets by Addition of Y_2_O_3_

**DOI:** 10.3390/ma15155160

**Published:** 2022-07-25

**Authors:** Zhuang Liu, Chaoyue Zhang, Haichen Wu, Renjie Chen, Aru Yan

**Affiliations:** 1 CISRI & NIMTE Joint Innovation Center for Rare Earth Permanent Magnets, Ningbo Institute of Materials Technology and Engineering, Chinese Academy of Sciences, Ningbo 315201, China; 2 University of Chinese Academy of Sciences, Beijing 100049, China

**Keywords:** Sm_2_Co_17_ type magnets, high temperature magnetic properties, Y_2_O_3_ addition, strong pinning field, cellular structure

## Abstract

In this study, the effects of Y_2_O_3_ addition on the magnetic properties, microstructure and magnetization reversal behavior of Sm(Co_0.79_Fe_0.09_Cu_0.09_Zr_0.03_)_7.68_ magnet were investigated. By addition of Y_2_O_3_, the coercivity was increased from 21.34 kOe to 27.42 kOe at 300 K and from 5.14 kOe to 6.27 kOe at 823 K. A magnet with a maximum magnetic energy product of 9.86 MGOe at 823 K was obtained. With the interdiffusion of Y and Sm after appropriate addition, the Cu content within the cell boundary phase close to the oxide was detected to be nearly twice as high as that away from the oxide. We report for the first time that a collection of lamellar phases were formed on both sides of the inserted oxide, providing a strong pinning field against magnetic domain wall motion based on in-situ Lorentz TEM observation. Furthermore, the ordering process of the original magnet was delayed after Y_2_O_3_ addition, resulting in the refinement of cellular structure, which can also enhance the domain wall pinning ability of cellular structures based on micromagnetic simulation. However, excessive addition of Y_2_O_3_ led to large Cu-rich phase and Zr-rich impurity phase precipitated at the edge of the oxide, resulting in the destruction of cellular structures and a significant reduction in coercivity. This study provides a new technical approach to regulate the microstructure of Sm_2_Co_17_ type magnets. Addition of Y_2_O_3_ is expected to play a significant role in improvement of high temperature magnetic properties.

## 1. Introduction

Sm_2_Co_17_ type sintered magnets are known for their high curie temperature and excellent thermal stability [1]. These characters make them the best choice for applications with ambient temperatures above 573 K [2,3,4]. They are widely used in microwave tubes, gyroscope, sensors and accelerometers to control and stabilize satellites, magnetic bearings, and actuators [5]. The enhancement of high temperature magnetic properties will play a more significant role in improving the performance of these types of equipment. As we know, cellular structure plays an important role in the Sm_2_Co_17_ type of sintered magnet. It consists of Fe-rich 2:17R (Th_2_Zn_17_) cell phase, Cu-rich 1:5 (CaCu_5_) cell boundary phase, and Zr-rich 1:3 lamellar phase, which are essential to the magnetic properties [6,7,8]. The cell phase contributes to high saturation magnetization and remanence, while the 1:5 Cu-rich cell boundary phase and the 1:3 Zr-rich lamellar phase act as the pinning center against the domain wall movement [9,10,11]. The cellular structure evolves from the metastable solution precursor obtained by sintering at 1453–1503 K and solid solution treated at 1423–1473 K, followed by quenching treatment. The solution precursors gradually form cellular structures after aging treatment through the ordering phase transformation and segregation of the elements [12,13,14,15,16,17].

For Sm_2_Co_17_ type sintered magnets, coercivity is the key factor affecting magnetic properties at high temperature, which is dominated by domain wall pinning. The coercivity of Sm_2_Co_17_ type sintered magnets is closely related to the difference in the domain wall energy between 2:17R cell phase and 1:5 cell boundary phase ($\Delta \gamma_{2:17/1:5}$), which is determined by the Cu and Fe content in 2:17R cell phase and 1:5 cell boundary phase. Generally speaking, the larger of |$\Delta \gamma_{2:17/1:5}$|, the higher the coercivity acquired. In high-temperature resistant magnets, higher content of Cu in the cell boundary phase is favorable for coercivity both at room temperature and high temperature. According to the reports of G.C. Hadjipanayis et al. [18], the effects of composition (Cu, Fe, Zr, Cu, Sm) and processing (aging temperature and time) on magnetic properties may be summed up as changing microstructure, which leads to a variation of the distribution and content of Cu at the 1:5 cell boundaries. Thus, in order to obtain high coercivity at high temperature, the traditional way is to include a large amount of Cu in Sm_2_Co_17_ type magnets. However, this reduces the remanence and magnetic energy product at room and high temperature quickly, as shown in the research results of Liu et al. [19]. Up to now, different Sm_2_Co_17_ type magnets with high temperature resistance have been developed. M. S. Walmer et al. [3] first reported a new class of Sm_2_Co_17_ type sintered magnets with a magnetic energy product of 6.3 MGOe at 550 °C, and N. J. Yu et al. [20] reported the highest magnetic energy product of 11.9 MGOe at 500 °C. After much research into composition and heat treatment processes, the high temperature coercivity and magnetic energy product of Sm_2_Co_17_ type sintered magnets have reached a bottleneck, and any improvement is extremely difficult.

In recent years, however, there are some new approaches that can also greatly enhance the coercivity of Sm_2_Co_17_ type sintered magnets. Wang et al. [21] reported that fine Cu powder addition induced a coercivity enhancement. Yan et al. [22] tried CuO doping by the dual alloy method in Sm_2_Co_17_ type magnets and obtained a clear increase of coercivity by eliminating the Cu-lean phenomenon at the grain boundary in Sm_2_Co_17_ type magnets. Furthermore, Wang et al. [23] reported that the coercivity of Sm_2_Co_17_ type magnets can also be enhanced by doping with a small amount of La_2_O_3_, which resulted in a higher strength of magnetic domain wall pinning nearby the oxide. Therefore, if we can use the great enhancement of coercivity in Sm_2_Co_17_ type magnets froma adding a small amount of rare earth oxide rather than a large amount of Cu, it could be possible to further improve the high-temperature magnetic properties of Sm_2_Co_17_ type magnets. In view of the higher saturation magnetization for the Y_2_Co_17_ compound compared to the Sm_2_Co_17_ compound, Y_2_O_3_ could be a better doping choice to achieve this by avoiding the degradation of magnetic properties due to mutual diffusion between the oxide and matrix phase.

In this work, a small amount of yttrium oxide (Y_2_O_3_) is added to Sm(Co_0.79_Fe_0.09_- Cu_0.09_Zr_0.03_)_7.68_ magnet with the purpose of enhancing room temperature as well as high temperature coercivity. Results show clearly that the coercivity at room temperature and high temperautre can both be enhanced, with better magnetic properties obtained at 823 K. The elements distribution and cellular structure were investigated in detail from solution precursor to aged magnet, especially for the area around the doped oxide. Different cellular structures are observed in the matrix phase after this addition. The coercivity improvements are clarified by in-situ analysis of domain wall movement under an applied magnetic field.

## 2. Materials and Methods

An ingot with nominal composition of Sm(Co_0.79_Fe_0.09_Cu_0.09_Zr_0.03_)_7.68_ was prepared by induction melting and crushed into ~3.5 μm powder by coarse crushing and jet-milling. Then, Y_2_O_3_ particles with an average size of 4.0 μm and 99.99% purity were blended into the prepared powders with amounts of 0 wt.%, 0.5 wt.%, 1 wt.%, 2 wt.%, 3 wt.% amount, respectively. The mixtures were oriented in a magnetic field of 2 T, and then isostatically compacted under a pressure of 160 MPa. The green compacts were sintered at 1503 K with a sintering time of 0.5 h and were solution-treated at 1463 K for 3 h under an argon atmosphere. Then, the green magnets were subjected to isothermal aging at 1103 K for 12 h, followed by slow cooling to 673 K at a rate of 0.7 K/min and holding for 3 h at 673 K. They were finally quenched to room temperature.

The microstructure and microchemistry of these samples were characterized using a field emission scanning electron microscope (SEM manufactured by Hitachi Limited, Tokyo, Japan) with energy dispersive spectrometer (EDS) detector, a Talos F200X transmission electron microscope (TEM manufactured by ThermoFisher, Waltham, MA, USA) with an acceleration voltage of 200 kV and equipped with energy dispersive X-ray spectroscopy (EDS) detectors and JEM-2100F Lorentz TEM microscope(manufactured by JEOL, Tokyo, Japan). The finite element micromagnetic simulations based on the Landau–Liftshitz–Gilbert–Langevin equations were performed to simulate the magnetization reversals process for different cellular structures. The magnetic properties of these samples were measured using a SQUID-VSM magnetic property measurement system (MPMS manufactured by Quantum Design, San Diego, CA, USA) under a magnetic field up to 7 T. Magnetization curves were collected at temperatures of 300 K, 373 K, 473 K, 573 K, 673 K, 773 K and 823 K, respectively.

## 3. Results and Discussion

### 3.1. Magnetic Properties

**Figure 1:**
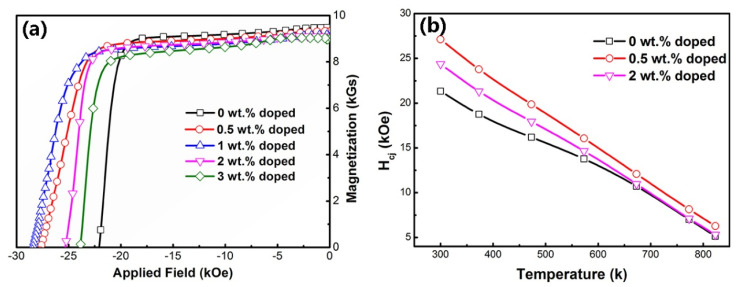
The demagnetization curves of magnets doped with different amounts of Y_2_O_3_ at room temperature (**a**); the H_cj_ variation of magnets with 0 wt.%, 0.5 wt.% and 2 wt.% addition from 300 K to 823 K (**b**).

Figure 1a shows the demagnetization curves of magnets with different amounts of added Y_2_O_3_ (0 wt.%, 0.5 wt.%, 1 wt.%, 2 wt.% and 3 wt.%). Table 1 shows the variation of the B_r_, H_cj_ and (BH)_max_ from 300 K to 823 K as a function of the Y_2_O_3_ addition. At room temperature, the B_r_ monotonically decreases from 9.45 kGs to 9.02 kGs and (BH)_max_ shows small decreases from 20.97 MGOe to 19.48 MGOe with the increase of Y_2_O_3_. However, H_cj_ increases dramatically from 21.34 kOe to 27.13 kOe when the addition amount is 0.5 wt.% and further increases to 27.42 kOe with the 1 wt.% mixture. It then decreases to 24.37 kOe and 21.43 kOe when the addition amount reaches 2 wt.% and 3 wt.%, respectively. With temperature increase, the B_r_, H_cj_ and (BH)_max_ all show a monotonically decreasing variation, while the hybrid magnets show higher magnetic properties at high temperature. Clearly, the monotonic reduction of remanence is due to the addition of nonmagnetic oxides. The remanence will be further reduced as temperatures increase due to thermal magnetic attenuation. Here, interesting changes of coercivity for the original magnet and hybrid magnets with increasing temperature are shown in Figure 1b. For the original magnet, the coercivity decreases slowly with the increase of temperature up to 573 K, while it decreases rapidly as temperature increases further. This has also been reported in some other research results for high-temperature resistant Sm_2_Co_17_ type magnets [24,25]. However, with 0.5 wt.% additon of Y_2_O_3_, this tendency changes to a linear reduction by enhancing the coercivity at room temperature. Higher coercivity is obtained at temperatures up to 823 K, which is about 1.13 kOe higher than the original magnet. As the addition amount further increases to 2 wt.%, although the coercivity at temperatures lower than 573 K is improved, it is barely enhanced at higher temperatures. This indicates that the enhancement of coercivity at high temperature comes from the great improvement of coercivity at room temperature by minor addition of Y_2_O_3_. Due to the improvement of high temperature coercivity, excellent high-temperature magnetic properties are achieved in magnets with 0.5 wt.% addition: remanence B_r_ = 8.39 kGs, coercivity H_cj_ = 16.07 kOe, magnetic energy density (BH)_max_ = 16.36 MGOe at 573 K and remanence B_r_ = 6.78 kGs, coercivity H_cj_ = 6.27 kOe, magnetic energy density (BH)_max_ = 9.86 MGOe at 823 K.

### 3.2. Analysis of Elements’ Diffusion, Cellular Structure and Phases

**Figure 2:**
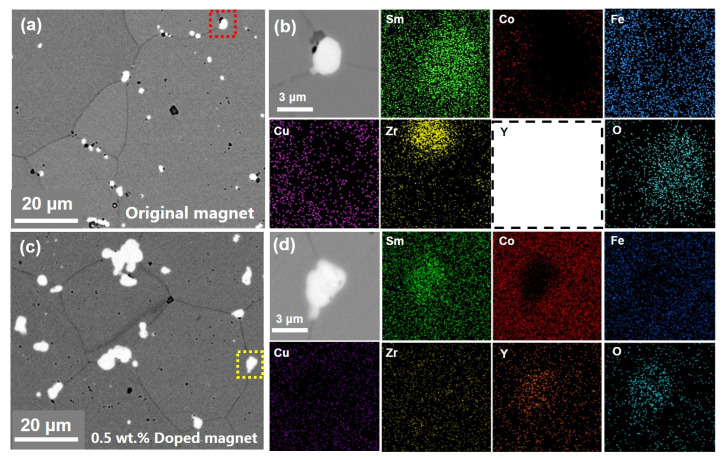
The SEM image of the original magnet (**a**) and EDS mapping of magnified region marked by a red dotted box (**b**), the SEM image of 0.5 wt.% doped magnet (**c**) and EDS mapping of magnified region marked by a yellow dotted box (**d**).

**Figure 3:**
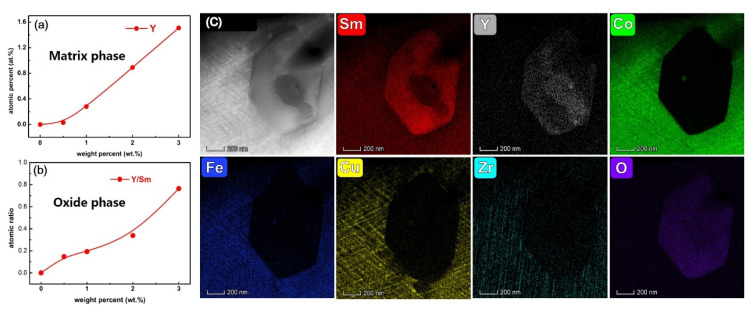
The content of Y in matrix phase (**a**) and the atomic ratio of Y and Sm in the oxide (**b**), HAADF images (**c**) and their EDS elemental distributions for the matrix phase around the Sm-Y oxide in magnets with 1 wt.% addition.

In order to analyze the reasons for the improvement of coercivity, the distribution of elements was investigated. Figure 2 show the SEM images of the original magnet and a magnet with 0.5 wt.% addition and their EDS elemental distributions of Sm, Co, Fe, Cu, Zr, Y and O. It can be seen that all the magnets contains three phases: the grey matrix phase, the white oxides phase and very scarce dark Zr-rich phase. From the elemental mapping images, the oxides contain Sm, Y and O in the hybrid magnets, while only Sm and O are found in the oxides of the original magnet. This indicates that the added Y_2_O_3_ reacts with Sm element in the matrix phase. Sm goes into the oxide and replaces Y, while Y diffuses into the matrix phase. According to Figure 3a,b, as the amount of Y_2_O_3_ increases, the content of Y in the matrix phase increases and the atomic ratio of Y and Sm in the oxide phase also increase from 14.8% to 76.4%, which means lesser amounts of Y are replaced by Sm in the oxide phase compared with the matrix phase. Yhe inadequately diffused oxides were clearly observed in the HAADF images of magnets with 1 wt.% addition and their EDS elemental distributions is shown in Figure 3c. It can seen that the cellular structure is well formed around the oxide. At the core region of the oxide, a higher content of Y still exists, while more content of Sm than Y is observed in peripheral regions of the oxide. Almost no content of other elements are found in the oxide, which indicates that the content of Co, Fe, Cu, Zr elements in the matrix phase are not affected by the addition of Y_2_O_3_.

**Figure 4:**
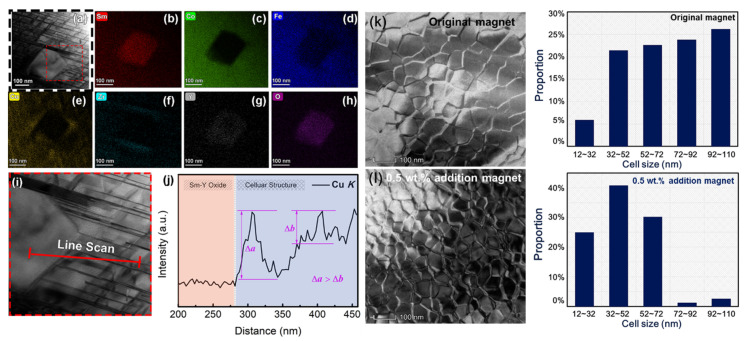
The TEM bright field image of 2:17R cell phase, 1:5 cell boundary phase and Zr-rich lamellar phase with an oxide inserted (**a**) and its EDS mapping (**b**–**h**), the magnified Cu-K line scan result (**j**) beginning from matrix phase and going across the oxide (**i**), TEM bright field image for the cellular structure perpendicular to the c-axis for the original magnet (**k**) and magnet with 0.5 wt.% addition (**l**), showing the corresponding cell size distribution for the original magnet and hybrid magnet.

To study the influence on the microstructure of the addition of Y_2_O_3_, two magnets (original magnet, magnet with 0.5 wt.% addition) were chosen for further analysis. We investigated the matrix phase around the Sm-Y oxide by TEM. Figure 4a is a typical TEM bright field image of 2:17R cell phase, 1:5 cell boundary phase and Zr-rich lamellar phase with an oxide inserted. According to the EDS mapping results shown in Figure 4b–h, the oxide is enriched in Sm and Y but poor in Fe, Co, Cu and Zr, which is consistent with the SEM-EDS mapping results. More precisely, it is interesting to note that the density of the Zr-rich lamellar phase at the tangency of the oxide is clearly higher than that in the matrix phase, which is more than 30 nm in width. The pile of the Zr-rich lamellar phases are also shown in the Zr-K mapping result. Accoring to the research results of Duerrschnabel [26], the intersections of lamellar and cellular phases are strong pinning centers of domain walls. It means these high density lamellar phases will show stronger pinning ability during the demagnetization process. In addition, from the Cu-K mapping, a Cu-rich layer (marked with red dotted box) is found to be circled around the oxide. To further study the uneven distribution of Cu, we demonstrate a line scan in STEM from the matrix phase and penetrating into the oxide, as is shown in Figure 4i,j. Clearly, there is a broader Cu-K content peak at the border of the oxide. As we know, in Sm_2_Co_17_ type magnets, the 1:5 cell boundary phase is rich in Cu while 2:17R cell phase is poor in Cu. Generally, the larger difference of Cu content in cell and cell boundary phase, the higher the domain wall energy difference and higher coercivity that can be achieved. The magnified Cu-K line scan result near the oxide in Figure 4j shows different variations of Cu content in the cell phase and cell boundary phase. The difference in Cu content ∆a (near the oxide) is about twice as large as ∆b (away from the oxide), indicating that the adjacent area near the oxide could provide a stronger magnetic domain wall pinning force. Figure 4h,k shows the TEM bright field images for the cellular structure perpendicular to c-axis for the original magnet and the magnet with 0.5 wt.% addition, respectively. The cellular structures of both magnets are uniform and complete, which is one of the key conditions for achieving high coercivity, indicating that adding an appropriate amount of Y_2_O_3_ will not destroy the typical cellular structure. Comparing the two magnets, the cell size of the hybrid magnet is smaller than that of the original magnet. The average cell size of the original sample is 71.93 nm. For a magnet with 0.5 wt.% addition, it decreases to 55.27 nm. This shows that the addition of Y_2_O_3_ can reduce the size of cellular structure.

According to the research of Liu et al. [19], an increase of Cu content in Sm(Co_bal_Fe_0.1_Cu_𝑥_Zr_0.033_)_7_ magnets can improve the coercivity at room temperature and 773 K, due to the higher Cu content in the Sm(CoCu)_5_ cell boundary phase. However, this is not the best way to enhance the magnetic properties at high temperature because of the decrease of saturated magnetization caused by adding too much Cu. Actually, some Cu does not go into the cell boundary phase, so that the beneficial influence of Cu is not used efficiently. Therefore, the best appproach is to improve the segregation of Cu in the cell boundary phase. However, the regulation of Cu segregation is very difficult in Sm_2_Co_17_ type magnets with a low content of Cu elements. According to our research results, the addition of rare earth oxide could be a new way to create higher Cu segregation without further increasing the Cu content.

**Figure 5:**
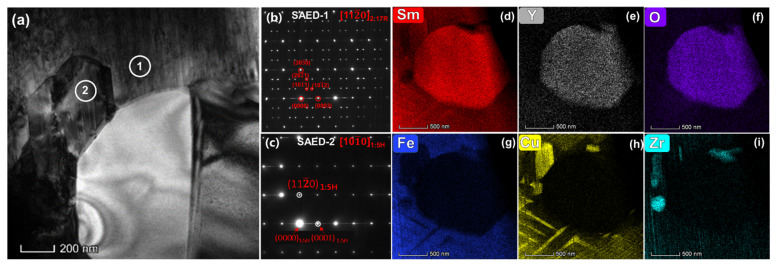
The TEM bright field image of cellular structure with the Y-Sm oxide inserted parallel to the c-axis (**a**) and its elements mapping (**d**–**i**), the selected area electron diffraction (SAED) of 2:17R cell phase (**b**) and the Cu-rich impurity phase (**c**).

In the hybrid magnet with 2 wt.% Y_2_O_3_ addition, the cellular structure parallel to c-axis characterized by TEM is shown in Figure 5. It can be seen that the cellular structure is formed around the Sm-Y oxide. Some thick cell boundary phases with higher Cu content are also observed near the oxide, which are similar with the results of 0.5 wt.% Y_2_O_3_ magnet; in contrast to the magnet with 0.5 wt.% addition, two impurity phases with about 250 nm wide precipitated at the edge of the oxide, which can be observed more clearly in STEM images with Cu-K (Figure 5h) and Zr-K (Figure 5i) mapping results. One is a Cu-rich phase mainly containing Sm, Co and Cu (marked with circle two in the TEM-BF image). In addition, a Zr-rich phase was formed adjacent to the Cu-rich phase and the oxide, which mainly contained Sm, Zr and Co elements. The selected area electron diffraction of 2:17R cell phase (SAED-1) and the Cu-rich impurity phase (SAED-2) are shown in Figure 4b and c, respectively. According to the orientation relation between 2:17R cell phase and 1:5H cell boundary phase [11$\bar{2}$0]_2:17R_//[10$\bar{1}$0]_1:5H_, the (0000), (0001), (11$\bar{2}$0), (11$\bar{2}$1) diffraction spots of the 1:5H cell boundary phase along [10$\bar{1}$0]_1:5H_ axis coincides with (0000), (0001), (30$\bar{3}$0), (30$\bar{3}$3) diffraction spots of 2:17R cell phase along [11$\bar{2}$0] axis. This relationship also can be seen in SAED-1 and SAED-2. Combined with the composition ratio of (Sm + Zr + Y):(Co + Fe + Cu) as 1:5.5, it could be inferred that the Cu-rich impurity phase is the same as the cell boundary phase. However, this large cell boundary phase is not distributed around the cell phase. According to the Cu content (~31.7 wt.%), it should be an weak magnetic phase. Thus, this large cell boundary phase cannot provide strong domain wall pinning fields and the antimagnetized domains tend to grow here, which is harmful for coercivity.

**Figure 6:**
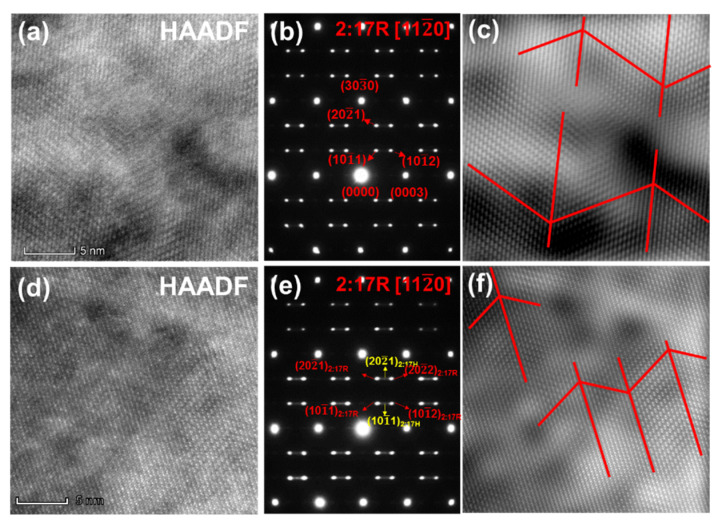
The original magnet isothermal aged for 1 min viewing along [11$\bar{2}$0]_2:17R_ (**a**) HRSTEM-HAADF, (**b**) SAED patterns, (**c**) inversed fast Fourier transformation (IFFT) images for the magnet with 0.5 wt.% addition isothermal aged for 1 min viewing along [11$\bar{2}$ 0]_2:17R_, (**d**) HRSTEM-HAADF, (**e**) SAED patterns, (**f**) IFFT images.

According to the recent research results of Wu et al. [17], the 2:17R cell phase and 1:5H cell boundary phase are formed through the ordering transformation of the disordered solution precursor during aging treatment. The microtwin structure that forms at the beginning of aging can lead to different cellular structures. Thus, the initial microstructure of ordering transformation is very important for the formation of cellular structure. Figure 6a–f shows high resolution STEM-HAADF images viewed along [11$\bar{2}$0]_2:17R_ and SAED patterns of the original solution precursor and solution precursor with 0.5 wt.% addition after 1 min isothermal aging followed by quenching. From STEM-HAADF images (Figure 6a,d), in the direction along the base plane, the atoms are arranged regularly as…-Sm-Sm-(Co-Co dumbbell)-Sm-Sm-(Co-Co dumbbell)-…. Along the direction perpendicular to the basal plane, they exhibit the stacking sequence of …ABCABC…, which is same as the characteristic stacking mode of the 2:17R phase, indicating that most of the solution precursor has undergone ordering transformation and formed 2:17R phase. The characteristic diffraction spots of 2:17R phase are shown in Figure 6b,e for the original magnet and hybrid magnet, respectively. As we know, ($10\bar{1}1$)_2:17H_ and ($20\bar{2}1$)_2:17H_ are the characteristic diffraction spots of 2:17H phase. In the hybrid magnet, two weaker characteristic diffraction spots of 2:17H phase are observed, indicating that a small amount of 2:17H phases are still retained. This means that the diffraction pattern of the cell phase is the superposition of diffraction patterns along the [11$\bar{2}$0]_2:17R_ zone axis of 2:17R phase and 2:17H phase. However, only characteristic diffraction spots of 2:17R phase are shown in the original magnet. The characteristic diffraction spots of 2:17R phase are elongated clearly in the hybrid magnet, which indicates that there are a large number of 2:17R ordered microregions in the initial ordering stage, forming a large number of stacking faults. According to the inversed fast Fourier transformation (IFFT) images (Figure 6c,f), the width of the 2:17R microtwin ordering region of the original magnet is about 5~10 nm, while it is less than 5 nm for the hybrid magnet. Therefore, it can be concluded that a small amount of disordered 2:17H phase and a short-range ordered 2:17R microregion are formed in the initial ordering stage for the hybrid magnet, while the ordering transformation has basically been completed at this stage for the original magnet, forming 2:17R phase with higher order degree. It means that the addition of Y_2_O_3_ delayed the ordering process of the original magnet, which could be the reason for the refinement of cellular structure in the hybrid magnet.

### 3.3. Analysis of Antimagnetization Behavior

**Figure 7:**
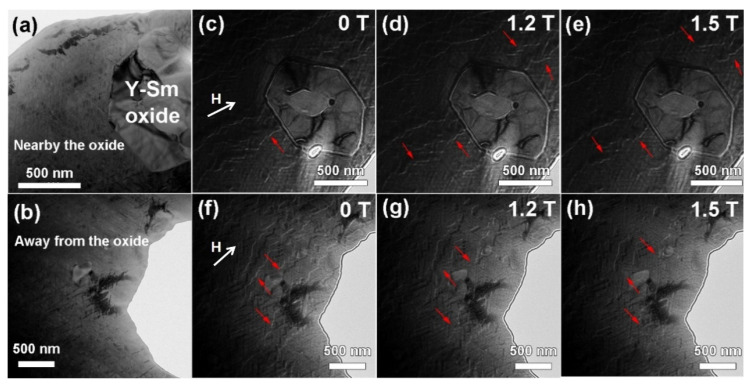
Fresnel Lorentz images for (**a**,**c**–**e**) the matrix nearby the Sm-Y oxide and (**b**,**f**–**h**) away from it in magnets with 0.5 wt.% Y_2_O_3_ addition at remanence states after external fields of 0 T, 1.2 T and 1.5 T.

A key question is how each of these differences of cellular structure influence the antimagnetization behavior, pinning strength and the coercivity. As we know, the coercivity of Sm_2_Co_17_ type magnets comes from the pinning effect of the cell boundary phase against the domain wall movement. The pinning force is closely related to the Cu and Fe content in cell boundary phase and cell phase. We utilized Lorentz TEM to demonstrate the magnetic domain wall movement and its pinning site in the matrix phase where a Sm-Y oxide is inserted. Figure 7a,b shows the TEM-BF image of a doped magnet near the oxide and away from the oxide with c-axis within the image plane. The corresponding EDS mapping and Fresnel Lorentz images are shown in Figure 7c–h. The white phase inserted in the matrix phase is a Sm-Y oxide. It is surounded by the cellular structure. The domain walls show zigzag shape and are pinned by a 1:5 cell boundary phase in the matrix phase, which is typical in Sm_2_Co_17_ type magnets. Some of the domain walls are cut off by the Sm-Y oxide due to its non-magnetic character. A magnetic field of 1.2 T and 1.5 T is applied on the sample outside TEM along the direction of c-axis indicated by the white arrows. Then the remanence states are observed by L-TEM as shown in Figure 7c–h. Some of the domain walls, which are away from the oxide, show depinning and move along the direction perpendicular to the c-axis. However, domain walls closer to the oxide show different behavior. Part of the domain wall near the oxide show still and are pinned, indicating that a stronger pinning force exists near the Sm-Y oxide. According to the result of the EDS mapping and the higher Cu content in cell boundary phase near the oxide, we can assume that the cell boundary phase near the oxide provides this stronger pinning field.

**Figure 8:**
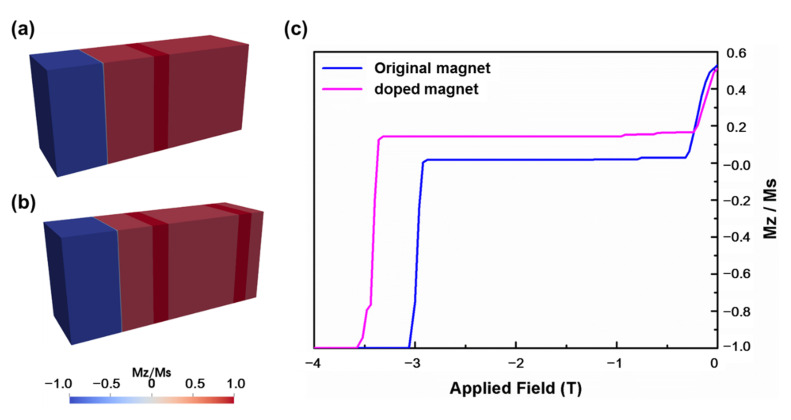
(**a**) Micromagnetic model with a cell boundary, (**b**) micromagnetic model with 2 cell boundaries, (**c**) theoretical demagnetization curves.

In order to study the effect of refinement of cellular structure on the domain wall movement and coercivity, two cellular structures with different numbers of cell boundary phases were built by micromagnetic simulation. The content of elements in the cell boundary phase and cell phase of the original magnet and the magnet with 0.5 wt.% addition was analyzed by EDS, and the average data are shown in Table 2. Here, the effect of Cu on the magnetic parameters of cell phase is ignored for low content. The effect of Fe is also ignored for same content in cell phases of both magnets. The effect of Fe on the magnetic parameters of cell boundary phase is also ignored due to low content. Then, the composition and corresponding Ms, K_1_ and A are used according to Lectard’s research results for Sm(Co_1-x_Cu_x_)_5_ compound [27]. Figure 8a, b shows the cellular structure with two cell phases sandwiched by one cell boundary phase and two cell boundary phases, respectively. Figure 8c,d shows the results of the simulated demagnetization curves. It can be seen that the cellular structure with two cell boundary phases obtained higher coercivity than that with one cell boundary phase, indicating that the refinement of cellular structure is also an important reason for the improvement of coercivity.

Based on the microstructure and magnetic domain analysis of the original magnet and the hybrid magnet, the increased coercivity resulted from Sm-Y oxide surrounded by complete cellular structure with higher Cu content in the cell boundary phase and high density lamellar phase. The addition of Y_2_O_3_ can also result in the refinement of the cellular structure. However, with increase of the amount added, some precipitation phase with weak magnetic properties formed near the oxide, which is harmful for coercivity. The formation of these defects may decrease the coercivity, the more so as the amount of Y_2_O_3_ added is increased.

## 4. Conclusions

High-temperature resistant Sm_2_Co_17_ type magnets with different content of added Y_2_O_3_ were prepared. The coercivity first increases and then decreases with the increase of added Y_2_O_3_, while the remanence decreases monotonically. The magnet with 0.5 wt.% Y_2_O_3_ addition has the best magnetic properties, with coercivity increasing by 5.79 kOe, 2.30 kOe and 1.13 kOe at 300 K, 573 K and 823 K, respectively. Therefore, the highest magnetic energy product of about 9.86 MGOe at 823 K was achieved. The mutual diffusion of Sm and Y between the oxide and the matrix during sintering and solid solution treatment lead to a gradual increase in the content of Y in the matrix and a higher content of Sm in the oxide. Almost no other elements are contained in the Sm-Y oxide. In magnets with 0.5 wt.% addition of Y_2_O_3_, the pile of the Zr-rich lamellar phase and cell boundary phase with higher content of Cu are formed near the oxide, resulting in a stronger pinning field and a significant increase in the coercivity at room temperature and high temperatures. Furthermore, the oxide addition reduces the order degree of the initial microstructure during ordering transformation and refines the cellular structure.

## Figures and Tables

**Table 1:** The magnetic properties of magnets doped with different amounts of Y_2_O_3_ from 300 K to 823 K.

Addition Amount		300 K	373 K	473 K	573 K	673 K	773 K	823 K
0 wt.%	B_r_ (kGs)	9.45	9.17	8.81	8.31	7.65	6.72	6.26
	H_cj_ (kOe)	21.34	18.76	16.19	13.77	10.72	7.01	5.14
	(BH)_max_ (MGOe)	20.97	19.71	17.93	15.92	13.04	9.87	7.79
0.5 wt.%	B_r_ (kGs)	9.35	9.13	8.85	8.39	7.90	7.25	6.78
	H_cj_ (kOe)	27.13	23.78	19.87	16.07	12.09	8.15	6.27
	(BH)_max_ (MGOe)	20.61	19.82	18.3	16.36	14.27	11.64	9.86
1 wt.%	B_r_ (kGs)	9.19	8.89	8.60	8.06	7.45	6.66	6.22
	H_cj_ (kOe)	27.42	23.81	19.65	15.41	10.78	6.27	4.49
	(BH)_max_ (MGOe)	19.71	18.59	17.05	15.14	12.54	9.64	7.52
2 wt.%	B_r_ (kGs)	9.10	8.84	8.36	7.89	7.26	6.60	6.24
	H_cj_ (kOe)	24.37	21.32	17.95	14.65	10.97	7.11	5.32
	(BH)_max_ (MGOe)	19.51	18.57	16.54	14.39	11.94	9.64	7.72
3 wt.%	B_r_ (kGs)	9.02	8.90	8.52	8.16	7.62	6.68	6.22
	H_cj_ (kOe)	21.43	18.88	16.25	13.72	10.64	7	5.14
	(BH)_max_ (MGOe)	19.48	18.95	16.86	15	12.77	9.72	7.59

**Table 2:** Composition corresponding to the cell phase and cell boundary phase in the original magnet and the magnet with 0.5 wt.% addition and its K_1_, M_s_ and A.

Phase		Composition	K_1_ (MJ/m^3^)	M_s_ (T)	A (pJ/m)
Cell phase		Sm_2_Co_17_	4.2	1.22	14
Cell boundary phase	original magnet	Sm(Co_0.7_Cu_0.3_)_5_	8.7	0.56	6.6
	magnet with 0.5 wt.% addition	Sm(Co_0.72_Cu_0.28_)_5_	9.36	0.588	6.8

## Data Availability

Not applicable.
